# Extreme Depletion of PIP_3_ Accompanies the Increased Life Span and Stress Tolerance of PI3K-null *C. elegans* Mutants

**DOI:** 10.3389/fgene.2013.00034

**Published:** 2013-03-28

**Authors:** Puneet Bharill, Srinivas Ayyadevara, Ramani Alla, Robert J. Shmookler Reis

**Affiliations:** ^1^McClellan VA Medical Center, Central Arkansas Veterans Healthcare SystemLittle Rock, AR, USA; ^2^Department of Biochemistry and Molecular Biology, University of Arkansas for Medical SciencesLittle Rock, AR, USA; ^3^Department of Geriatrics, University of Arkansas for Medical SciencesLittle Rock, AR, USA

**Keywords:** insulin signaling, IGF-1, longevity, oxidative stress, PI3K, phosphatidylinositides, *C. elegans*/nematode

## Abstract

The regulation of animal longevity shows remarkable plasticity, in that a variety of genetic lesions are able to extend lifespan by as much as 10-fold. Such studies have implicated several key signaling pathways that must normally limit longevity, since their disruption prolongs life. Little is known, however, about the proximal effectors of aging on which these pathways are presumed to converge, and to date, no pharmacologic agents even approach the life-extending effects of genetic mutation. In the present study, we have sought to define the downstream consequences of *age-1* nonsense mutations, which confer 10-fold life extension to the nematode *Caenorhabditis elegans* – the largest effect documented for any single mutation. Such mutations insert a premature stop codon upstream of the catalytic domain of the AGE-1/p110α subunit of class-I PI3K. As expected, we do not detect class-I PI3K (and based on our sensitivity, it constitutes <14% of wild-type levels), nor do we find any PI3K activity as judged by immunodetection of phosphorylated AKT, which strongly requires PIP_3_ for activation by upstream kinases, or immunodetection of its product, PIP_3_. In the latter case, the upper 95%-confidence limit for PIP_3_ is 1.4% of the wild-type level. We tested a variety of commercially available PI3K inhibitors, as well as three phosphatidylinositol analogs (PIAs) that are most active in inhibiting AKT activation, for effects on longevity and survival of oxidative stress. Of these, GDC-0941, PIA6, and PIA24 (each at 1 or 10 μM) extended lifespan by 7–14%, while PIAs 6, 12, and 24 (at 1 or 10 μM) increased survival time in 5 mM peroxide by 12–52%. These effects may have been conferred by insulinlike signaling, since a reporter regulated by the DAF-16/FOXO transcription factor, SOD-3::GFP, was stimulated by these PIAs in the same rank order (PIA24 > PIA6 > PIA12) as lifespan. A second reporter, PEPCK::GFP, was equally activated (∼40%) by all three.

## Introduction

Phosphatidylinositides are tightly regulated signaling molecules that participate in a diverse range of cellular events, including cell replication and survival, membrane trafficking, secretion, adhesion, and cell migration (Boss and Im, 2012; Echard, 2012; Mayinger, 2012). Phosphatidylinositol (PI; sometimes abbreviated as “PtdIns”) lipid chains are generally integrated into inner cell membranes, while the attached phosphoinositide rings project into the cytoplasm. PI’s are formed by additions of phosphate to hydroxyl groups at the 1, 3, 4, and/or 5 position of the inositol ring. Additions at the 3 position are governed by phosphatidylinositol 3-kinases (PI3K’s), enzymes with key regulatory roles in cell division and metabolism (Wymann and Schultz, 2012). Class-I PI3K’s convert PI(4,5)P_2_ (often abbreviated as PIP_2_) to PI(3,4,5)P_3_ (or PIP_3_), which plays decisive roles in multiple signaling pathways.

Intracellular concentrations of l-α phosphatidylinositol 4,5-bisphosphate (known as PI(4,5)P_2_, one of several PIP_2_ isoforms) lie in the range of 2–30 μM (Gambhir et al., 2004). Class-I phosphatidylinositol 3-kinase (PI3K) can add phosphate to PI(4,5)P_2_ at the inositol 3 carbon to form phosphatidylinositol 3,4,5-triphosphate[PI(3,4,5)P_3_ or PIP_3_]. This key signaling molecule or “second messenger” is normally present at only ∼0.1% of the levels of its precursor (Weinkove et al., 2006), or <30 nM. In response to stimuli, however, the concentration of PIP_3_ can increase up to 100-fold (Pettitt et al., 2006), achieved through activation of PI3K and/or inactivation of the opposing PI 3-phosphatase, PTEN. Many membrane-associated proteins, including a number of kinases involved in signal transduction cascades, have a domain that binds either PIP_3_ or a specific PIP_2_. The best-studied of these are Pleckstrin Homology (PH) domains, typically ∼120 amino-acid residues long, many of which show quite specific affinity for PIP_3_. In the insulin signaling pathway, formation of PIP_3_ is required for the downstream activation of AKT/PKB, a protein kinase that promotes cell proliferation and blocks apoptosis in many cell types (Franke et al., 1997). In order to be activated, AKT must bind PIP_3_ at its PH domain. This tethers AKT to the inner cell membrane, in relative proximity to its upstream kinase(s) and downstream targets, while also inducing a structural change in AKT to expose a key phosphorylation site to activating kinases such as PDK-1 (Stokoe et al., 1997). Mammalian AKTs are fully active when phosphorylated at residues Thr^308^ and Ser^473^ (Stokoe et al., 1997); other AKT-activating kinases include DNA-dependent protein kinase (DNA-PK) (Dragoi et al., 2005; Sester et al., 2006) and TOR complex (Hawkins et al., 2006). Following dimerization, AKT1/AKT2 complex phosphorylates dozens of targets, including kinases and transcription factors, leading to their activation or inactivation (Cutillas et al., 2006). FOXO transcription factors (DAF-16 isoforms in *Caenorhabditis elegans*) are among the inactivated targets, as their phosphorylation by the AKT complex prevents their entry into the nucleus (Tissenbaum and Ruvkun, 1998; Berdichevsky et al., 2006). AKT mutations conferring constitutive activation are observed in many cancers (Shtilbans et al., 2008); mutations in the *pten* gene, disrupting the PI 3-phosphatase that opposes PI3K, also produce a high PIP_3_/PIP_2_ ratio, favoring activated AKT and hence cell proliferation in diverse cancers (Yi et al., 2005). Although direct constitutive activation of PI3K is far less common, the BCR-ABL fusion protein indirectly activates PI3K, thus elevating PIP_3_ in chronic myelogenous leukemia (Kharas et al., 2008).

The above findings demonstrate the critical involvement of PIP_3_/AKT/FOXO signaling in cell proliferation, and have led to great interest in disruption of such signaling in cancers (Castillo et al., 2004). However, this kinase cascade has also been implicated in other roles beyond cell proliferation. Enhanced PIP_3_ signaling in specific hypothalamic neurons is associated with diet-sensitive obesity (Plum et al., 2006). Lithium, commonly used as a mood stabilizer for bipolar disorder, suppresses PIP_3_ signaling in *Dictyostelium* and in cultured human cells (King et al., 2009). To date, attention has been largely focused on drugs that target PI3K or AKT. The two PI3K inhibitors in most common use are LY294002 and wortmannin (Vlahos et al., 1994; Schultz et al., 1995; Semba et al., 2002). These drugs bind to the ATP-binding site of PI3K, LY294002 reversibly (IC_50_ 0.5–10 μM) and wortmannin much more avidly (IC_50_ 7 nM)(Wu et al., 2008). Both are known to inhibit other kinases (e.g., PLK1) with similar IC_50_ values; in view of the thousands of proteins with ATP-binding sites, such off-target effects are not surprising. A number of PI3K inhibitors have been developed through small-molecule screens or by synthetic chemistry testing derivatives of partially effective molecules. Among these, ZSTK474 inhibits p110γ somewhat more than α or β (IC_50_’s of 6, 17, and 53 nM respectively); whereas A66 is rather specific for p110α (IC_50_ of 32 nM), requiring >3 μM to reach the IC_50_ against β or γ. The most avid p110α inhibitor is GDC-0941 with an IC_50_ of 3 nM, but its activity against other PI3K isoforms has not been reported. In another approach, phosphatidylinositol analogs (PIAs) were designed to dock in the PIP_3_-binding (PH) domain of AKT and thereby inhibit its activity (Kozikowski et al., 2003). It is not known whether any of these compounds have affinity for the PIP_2_-binding catalytic site of PI3K.

The *C. elegans age-1* gene encodes the nematode homolog of p110α, the mammalian class-I or -Iα catalytic subunit of PI3K (Morris et al., 1996) responsible for conversion of PIP_2_ to PIP_3_. Nonsense mutants of *age-1*, at the second homozygous generation, should lack any active PI3K. These worms are extremely long-lived and resistant to multiple stresses; they develop very slowly and are completely infertile, reflecting severely impaired cell division (Ayyadevara et al., 2008). Those phenotypes were largely blunted in the first homozygous generation, presumably due to carry-over of oocyte PI3K, *age-1* mRNA, or PIP_3_ from their *age-1*-heterozygous parent.

Because PIP_3_ can allosterically alter the conformation of a PH-domain protein, permitting its activation by one or more kinases, PIP_3_ may serve as a catalyst for that activation and be required only in minute amounts. In contrast, it is required continuously (and hence stoichiometrically) for its membrane-tethering role. We therefore predict that, over most of its physiological range of concentration, PIP_3_ will have an essentially linear dose-response curve with respect to activity of any individual PIP_3_-binding protein (although it would become non-linear for any process that depends on several such proteins). However, for binding proteins that also depend on PIP_3_ “catalytically,” such as AKT, a far more dramatic effect might be expected on removal of the last few PIP_3_ molecules per cell – perhaps accounting for the marked phenotypic differences between first- and second-generation *age-1*-null homozygotes (Ayyadevara et al., 2008).

We have now examined PIP_3_ levels by quantitative immunofluorescence as well as functional assays, in first and second-generation *age-1*(*mg44*) homozygotes, comparing them to worms that bear wild-type or weaker *age-1* mutant alleles. We also have asked whether PI3K inhibitors can partially “phenocopy” *age-1* mutation in wild-type animals, to enhance longevity and stress tolerance.

## Results

### PI3K is widely distributed in wild-type *C. elegans*, but is not detected over background in *age*-1-null mutant worms

**Figure 1:**
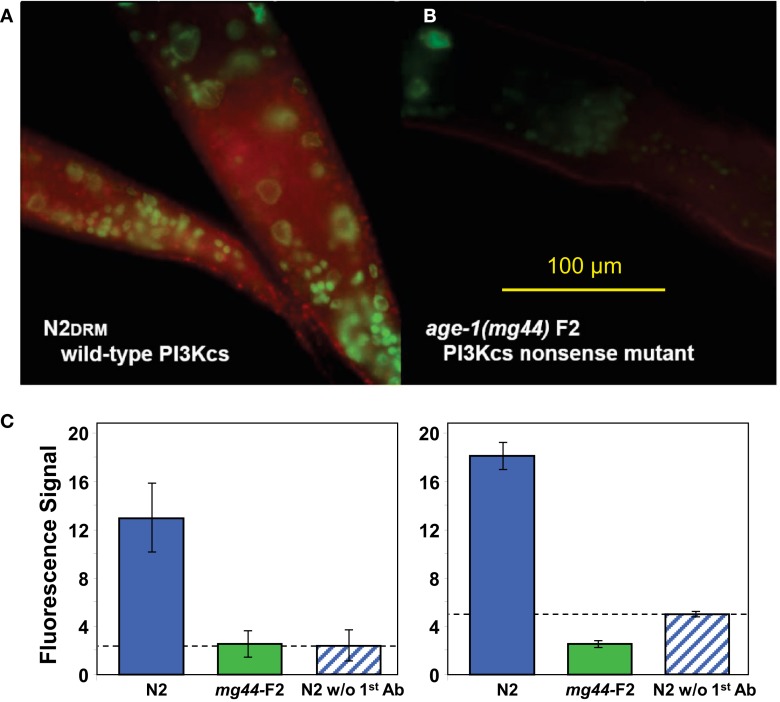
**Immunodetection of AGE-1 protein (class-I PI3K catalytic subunit) in adult *C. elegans* of wild-type strain N2DRM (A) or second-generation *age-1*(*mg44*) homozygotes at adult age 3 days (B)**. Synchronized worms, fixed 14 h in 1% formaldehyde at 4°C, were permeabilized by successive exposures to 1% β-mercaptoethanol, 10-mM DTT, and 0.3% hydrogen peroxide. Primary antibody was goat anti-AGE-1 at1:50 (Santa Cruz Biotech.), followed by rabbit anti-goat ALEXA680-tagged IgG at 1:200 (Invitrogen), imaged on an Olympus BX51 fluorescence microscope at 10×. The histograms **(C)** show mean fluorescence intensity, ±SEM, in two independent experiments. Each includes a negative control, staining of wild-type worms without primary antibody (rightmost bar).

The *age-1*(*mg44*) allele encodes a truncated PI3K p110α protein, due to replacement of the Trp codon at position 387 (of 1146) by an amber stop codon (Ayyadevara et al., 2008). Antibody raised to the helical and kinase domains of AGE-1, lying downstream of (C-terminal to) the mutation, registered full-length enzyme as a diffuse cytoplasmic signal in virtually all cell types of wild-type *C. elegans* adults (Figures 1A,C). The same antibody, however, failed to detect AGE-1 protein in second-generation *age-1*(*mg44*) homozygotes, over the background seen in wild-type worms exposed only to the fluorescent secondary antibody (Figures 1B,C).

### PIP_3_ is significantly reduced in first-generation *age*-1(*mg*44) homozygotes, and is below detectable limits in their second-generation progeny

**Figure 2:**
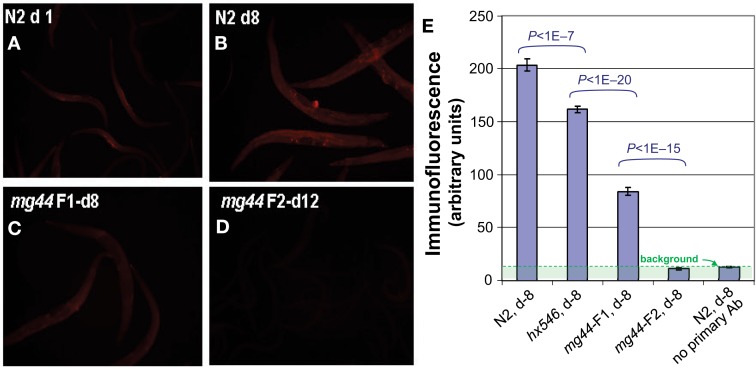
**Immunofluorescence quantitation of PIP_3_ in *C. elegans***. Worms were permeabilized as for Figure 1 and incubated with mouse antibody to PIP_3_ (Echelon), followed by a 1:200 dilution of secondary antibody, ALEXA594-labeled goat anti-mouse IgG (Invitrogen). Images **(A–D)**, acquired on an Olympus BX51 microscope, were quantified with ImageJ and summarized in **(E)** for a typical experiment. Histogram bars show means ± SEMs for 20–50 worms per group. Background, assessed without primary antibody, is shown (rightmost bar) but has not been subtracted. Worm ages are given in days (d) as adult.

Despite the absence of full-length, catalytically active AGE-1 protein, it is possible that PIP_3_ might be generated by a PI3K p110 of a different class, or *via* an alternative biosynthetic pathway. We therefore assessed PIP_3_ levels, initially and most sensitively by *in situ* immunofluorescence but with confirmation by activity-based assays. Using a highly specific antibody against PIP_3_ (Chen et al., 2002; Kharas et al., 2008), we found that *age-1*(*mg44*) second-generation homozygotes have no PIP_3_-specific immunofluorescence above background, i.e., their level is indistinguishable from negative control samples from which the primary antibody was omitted (Figure 2). In several replicate experiments comparing groups of day-8 adults (of which Figure 2 is typical), we observed 15–25% reductions in PIP_3_ signal for worms carrying the weaker *hx546* allele of *age-1* (each *P* < 0.001 compared to wild-type), 60–75% reductions for first-generation *mg44* homozygotes (each *P* < 10^−12^), and essentially no signal above background in second-generation *mg44*/*mg44* adults (*P* < 10^−12^ relative to any group except negative controls). Because the upper bound of the 95% confidence interval for these “F2” *mg44*^−/−^ worms is ∼1.4% of the wild-type level, we infer that their PIP_3_ level is reduced at least 70-fold relative to wild-type adults. PIP_3_ levels were also assessed at several ages. Wild-type N2 worms showed maximal signal at 3 days of adult age (coinciding with peak fecundity) and fell to 35–40% of maximum at 6–10 days, whereas second-generation *mg44* homozygotes never differed significantly from background at adult ages 3, 5, or 10 days (data not shown).

**Figure 3:**
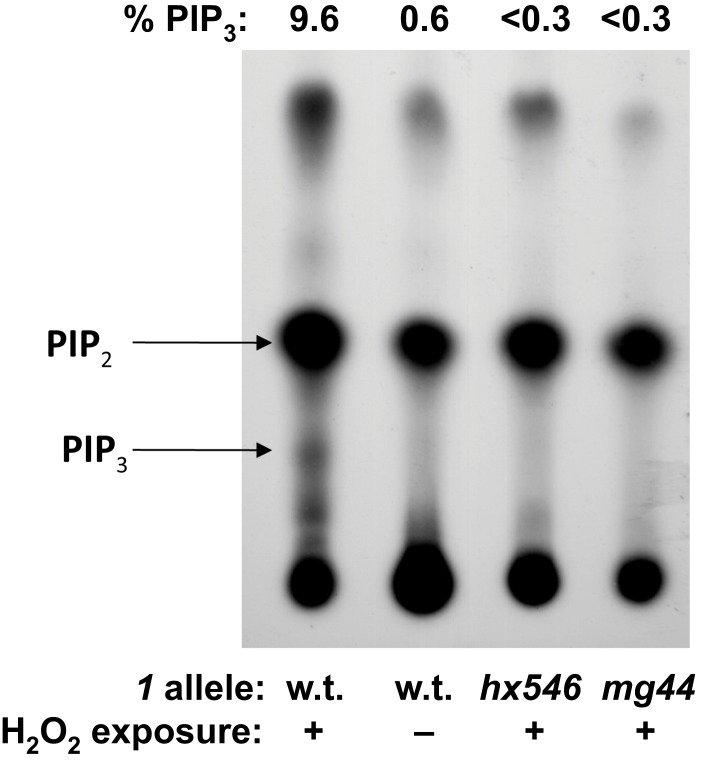
***In vivo*^32^P-labeling of PIP_2_ and PIP_3_**. Young adult worms were washed and incubated 16 h in phosphate-free medium to which 0.6 mCi ${\;}^{32}{\text{PO}}_{4}^{=}$ were added. Where indicated, worms were exposed 45 min to 16-mM H_2_O_2_; lipids were extracted, chromatographed, and autoradiographed. Quantitative results (PIP_3_ as a percent of the sum of PIP_2_ and PIP_3_), determined by scintillation counting of excised spots, are given above the TLC image.

Activity-based assays were used to confirm immunofluorescence quantitation of class-Iα PI3K, and of PIP_3_. PI3K activity is difficult to quantify in unstimulated cells, in which it is below the limits of detection, but it can be measured after induction by oxidative stress (Weinkove et al., 2006). We induced oxidative stress by exposing adult worms to 4 mM H_2_O_2_ for 40 min at 20°C, in the presence of ${\;}^{32}{\text{PO}}_{4}^{=}$. Worms were then lysed and their PIP’s isolated and resolved by thin-layer chromatography. PI3K activity was calculated as the ratio of ^32^P incorporation coinciding with the position of a PIP_3_ standard, to ^32^P signal migrating with PIP_2_. As expected, no PIP_3_ signal was detected in the absence of peroxide stress. After H_2_O_2_ exposure, it reached a measurable level (a ratio of 0.07) only for wild-type worms, but remained near-zero for worms carrying either *age-1* allele (Figure 3).

**Figure 4:**
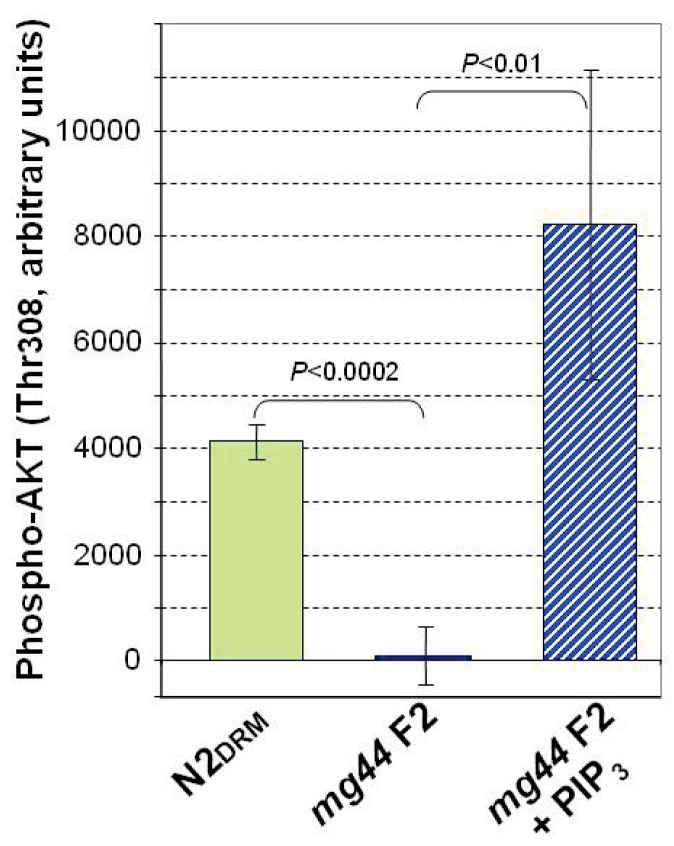
**Functional assay of PIP_3_ based on phosphorylation of AKT(Thr^308^)**. Bacterially synthesized AKT, with an N-terminal His_6_ tag, was added to cleared *C. elegans* lysates, and after 1 h at 20°C, was bound to AKT monoclonal antibody on AlphaBeads (PerkinElmer) in an AlphaScreen PI3K Assay (Echelon), and scored for also binding a tagged antibody to AKT(P-Thr^308^). Results were read on an Envision 2104 Microplate Reader (PerkinElmer). Error bars are standard deviations (*N* = 3).

Similarly, we were able to confirm PIP_3_ depletion in *age-1*(*mg44*) second-generation homozygotes, using a functional assay based on the requirement for PIP_3_-binding to bioactivate AKT *via* phosphorylation at Thr^308^ (Stokoe et al., 1997). Antibodies recognizing unphosphorylated human AKT, or specific for AKT phosphorylated at Thr^308^, were used to evaluate the products of incubating bacterially synthesized human AKT with *C. elegans* lysates (Figure 4). The results support our immunofluorescence data, indicating the virtual absence of any PIP_3_ in the very long-lived *mg44*-mutant worms, relative to wild-type. Dependence of the assay on endogenous PIP_3_ was demonstrated by adding synthetic PIP_3_ (Figure 4, rightmost bar).

### Nematode lifespan and peroxide resistance are modestly enhanced by several PI3K inhibitors, in particular phosphatidylinositol analogs

**Figure 5:**
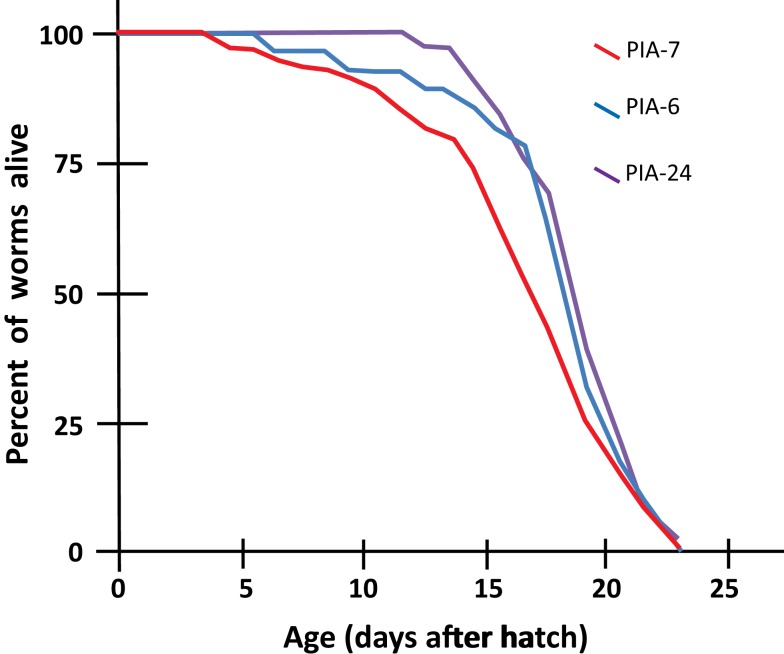
**Longevity survivals of wild-type (N2DRM) *C. elegans* maintained at 20°C on plates with phosphatidylinositol analogs (PIAs)**. Worms from synchronous cultures were selected as L4 larvae, and placed on 10-cm agar plates containing 1 μM of either PIA6, PIA24, or an inactive control, PIA7. They were transferred daily to identical plates on days 1–7 of adulthood to isolate them from their progeny, and transferred every 2–3 days thereafter. At each transfer, worms were scored as dead if they failed to move either spontaneously or in response to gentle prodding. Worms dying from desiccation due to stranding on the side walls of dishes, or from internal hatching of progeny, were censored at the midpoint of transfers between which death was discovered.

We tested a variety of PI3K inhibitors for the ability to extend nematode lifespan, in effect seeking a partial “pharmacopy” of the *age-1* phenotype. Neither wortmannin, LY294002, A66, nor ZSTK474 (each tested at 1–10 μM) increased the lifespan of wild-type worms (Table 1), However, GDC-0941 – reportedly the most avid p110α inhibitor – at 1 μM extended lifespan by 10% (nominally significant, Gehans–Wilcoxon *P* < 0.03). We then tested four phosphatidylinositol analogs (PIAs) previously shown to be potent inhibitors of signal transduction *via* AKT activation (Gills et al., 2006, 2007; Memmott et al., 2008). PIA6 and PIA24 increased adult life span in two of three repeats, relative to PIA7 (inactive control) or DMSO vehicle-treatment. In the experiment shown (Figure 5), mean longevity was extended 10% (*P* < 0.05) by PIA6, and nearly 14% by PIA24 (*P* < 0.015, Gehans–Wilcoxon log-rank test; Table 2).

**Table 1:** **Effects of PI3K inhibitors on wild-type lifespan at 25°C**.

	DMSO	LY294002	Wortmannin	A66	GDC-0941	ZSTK474
		**1 μM**	**1 μM**	**1 μM**	**1 μM**	**1 μM**
Mean survival (days)	10.72	10.51	10.28	9.78	11.64	10.96
SD	2.31	2.66	2.91	2.26	2.32	2.24
SEM	0.31	0.55	0.69	0.49	0.42	0.41
*N*	54	23	17	22	30	29
% Of DMSO control		98.0	95.9	91.2	109.6	102.2
*P*_,_ Gehans–Wilcoxon	–	–	–	–	<0.03	–
		**10 μM**	**10 μM**	**10 μM**	**10 μM**	**10 μM**
Mean survival (days)		9.80	10.83	9.97	10.97	9.60
SD		2.62	2.34	2.20	2.03	1.97
SEM		0.57	0.52	0.49	0.45	0.40
*N*		21	20	21	20	24
% Of DMSO control		91.4	101.0	93.0	102.3	89.6

**Table 2:** **Effects of PIAs on wild-type (N2) lifespan at 20°C**.

	DMSO	PIA7	PIA6	PIA24
		1 μM	1 μM	1 μM
**EXPERIMENT 1**				
Mean survival (days)	18.8	19.2	20.3	20.5
SD	2.8	2.7	1.8	2.3
SEM	0.63	0.56	0.36	0.47
*N*	19	24	26	24
% (DMSO + PIA7)/2	–	–	106.8	108.0
*P*_,_ Gehans–Wilcoxon	–	–	<0.10	<0.03
**EXPERIMENT 2**				
Mean survival (days)	17.3	16.6	18.2	18.8
SD	3.2	4.6	3.8	2.5
SEM	0.40	0.63	0.72	0.45
*N*	64	52	28	32
% (DMSO + PIA7)/2	–	–	109.9	113.5
*P*, Gehans–Wilcoxon	–	–	<0.06	<0.015
Combined Signif.:			<0.006	<0.0004

**Figure 6:**
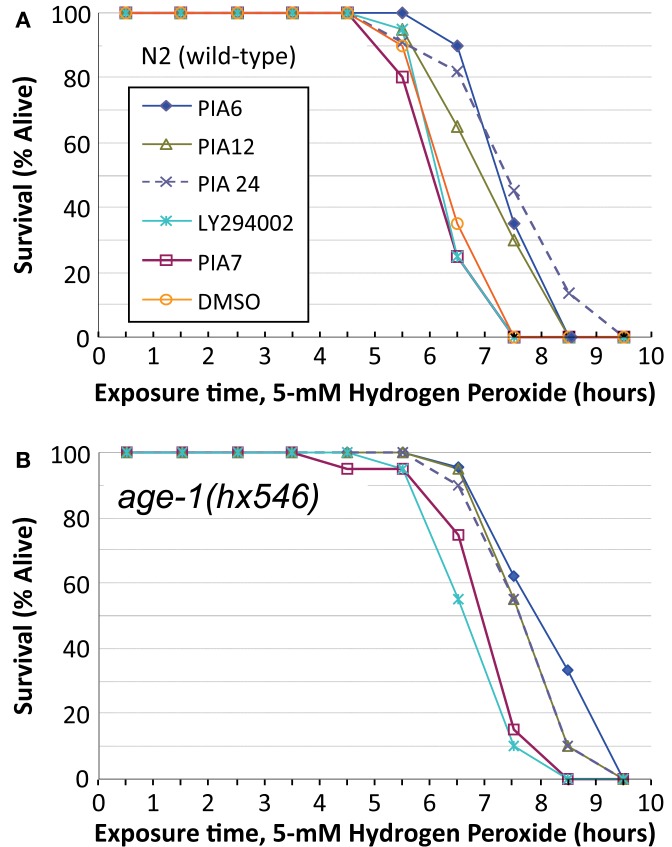
**Survival time during exposure to a toxic level of hydrogen peroxide, after development and growth in the presence of PIAs or LY294002**. Adult worms (day 2–3 after the L4/adult molt) of strain N2DRM [**(A)**, wild-type] or *age-1*(*hx546*) [**(B)**, a moderately long-lived *age-1* mutant] were maintained from hatching on 1 μM of LY294002 (a widely used inhibitor of class-I PI3K); PIA6, PIA12, or PIA24 (active PIP_3_ analogs); PIA7 (inactive control analog); or their common solvent, DMSO (added in the amount transferred with the inhibitors). Worms at adult day 3 were transferred to liquid medium without bacteria, containing 5 mM hydrogen peroxide, and were then monitored hourly for survival as described in the Figure 5 legend.

We then compared PIAs 6, 12, and 24 to LY294002 or an inactive PIA, for their ability to extend survival of *C. elegans* exposed to a lethal oxidative stress, 5 mM hydrogen peroxide. PIAs 6 and 24 again conferred the greatest protection to wild-type worms, extending survival by 18 and 19%, respectively (each *P* < 10^−6^), while PIA12 initially increased peroxide survival 12% (*P* < 0.003) and LY294002 by <1% (NS) relative to controls (Figure 6A; Tables 3). In these experiments, PIAs generally conferred less (and less significant) protection to *age-1*(*hx546*) worms, although PIA12 appeared equally effective for either strain (Figure 6B; Tables 3). When newly synthesized batches of PIAs 6 and 12 were subsequently tested (Tables 3), PIA12 was more protective than PIA6, indicating that differences in these analogs may in large measure reflect purity and freshness of the compounds. LY294002 was only moderately effective at doses ranging from 1 to 20 μM, whereas the tested PIAs remained equally protective from 1 to 1000 μM (Table 3 and additional data not shown).

**Table 3:** **Protection against 5-mM peroxide by PI3K inhibitors at 20°C**.

A. EXPERIMENT 1						
	DMSO	PIA7	LY294002	PIA6	PIA12	PIA24
		1 μM	1 μM	1 μM	1 μM	1 μM
**N2, wild-type**						
Mean survival (h)	6.8	6.6	6.7	7.9	7.5	8.0
SD	0.7	0.7	0.6	0.7	1.0	2.2
SEM	0.15	0.16	0.13	0.16	0.23	0.41
*N*	20	20	20	20	20	22
% of PIA7 control	–	–	101	118	112	119
*P*, Gehans–Wilcoxon	–	–	N.S.	<1E–6	0.002	<1E–6
***Age-1(hx546)***						
Mean survival (h)		9.8	10.8	10.0	11.0	9.6
SD		2.6	2.3	2.2	2.0	2.0
SEM		0.57	0.52	0.49	0.45	0.40
*N*		21	20	21	20	24
% of PIA7 control		–	96.5	116	112	112
*P*, Gehans–Wilcoxon		–	N.S.	0.0008	0.005	0.009

**B. EXPERIMENT 2**						
		**PIA7**	**LY294002**	**PIA6**	**PIA6 (10 μM) +**	
		**10 μM**	**10 μM**	**10 μM**	**LY294002 (10 μM)**	

**N2, wild-type**						
Mean survival (h)		6.0	7.1	7.1	7.5	
SD		1.0	1.5	1.1	1.4	
SEM		0.15	0.24	0.17	0.23	
*N*		40	40	40	40	
% of PIA7 control		–	118	118	125	
*P*, Gehans–Wilcoxon		–	<1E–3	<1E–5	<1E–6	
***Age-1(hx546)***						
Mean survival (h)		8.3	8.6	9.0	9.0	
SD		1.5	1.2	1.0	1.1	
SEM		0.23	0.19	0.16	0.17	
*N*		40	40	40	40	
% of PIA7 control		–	103	107	107	
*P*, Gehans–Wilcoxon		–	NS	<0.04	<0.05	

**C. EXPERIMENT 3**						
	**PIA7**	**PIA6**	**PIA12**		**PIA6 + LY**	**PIA12 + LY**
	**1 μM**	**1 μM**	**1 μM**		**1 μM, 10 μM**	**1 μM, 10 μM**

**N2, wild-type**						
Mean survival (h)	6.9	8.6	10.0		8.6	8.5
SD	1.6	1.6	1.1		1.7	1.7
SEM	0.25	0.25	0.17		0.26	0.27
*N*	40	40	40		40	40
% of PIA7 control	–	124	144		124	122
*P*, Gehans–Wilcoxon	–	<1E–5	<1E–12		<1E–4	<1E–4

**D. EXPERIMENT 4**						
	**PIA7**	**PIA6**	**PIA12**	**PIA7 + LY**	**PIA6 + LY**	**PIA12 + LY**
	**1 μM**	**1 μM**	**1 μM**	**1 μM, 10 μM**	**1 μM, 10 μM**	**1 μM, 10 μM**

**N2, wild-type**						
Mean survival (h)	6.2	7.3	9.4	6.4	7.4	7.4
SD	1.32	1.5	1.2	1.4	1.8	1.6
SEM	0.30	0.33	0.28	0.32	0.40	0.35
*N*	20	20	20	20	20	20
% of PIA7 control	–	118	152	103	119	118
*P*, Gehans–Wilcoxon	–	<0.02	<1E–8	0.65	<0.04	<0.02

### PIP_3_ analogs induce genes that are positively regulated by insulin/IGF-1 signaling

**Figure 7:**
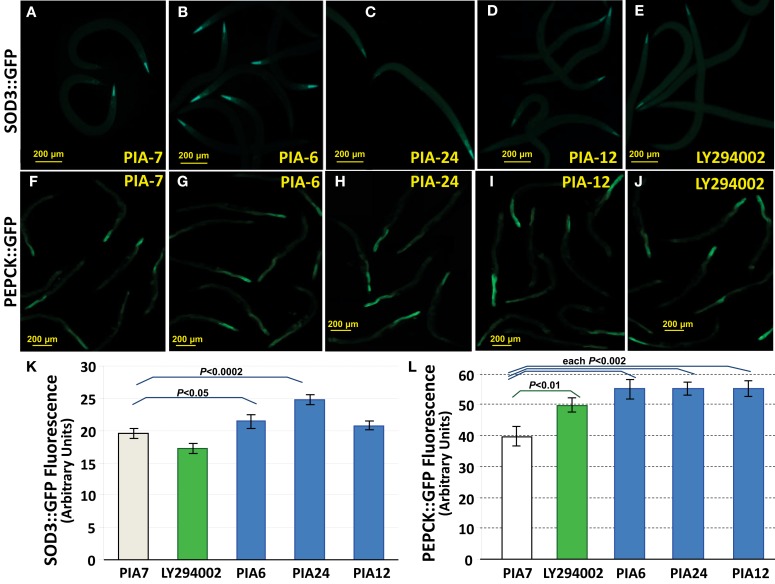
**PIAs induce elevated expression of known insulinlike signaling reporters, SOD-3:: GFP, and PEPCK::GFP**. Worms with integrated *sod-3::gfp* and *pepck::gfp* reporter constructs were grown 3 days in medium containing 1 μM PIA6, PIA12, or PIA24 (active PIP_3_ analogs); PIA7 (inactive control analog); or LY294002 (an inhibitor of class-I PI3K). Reporter fluorescence was then imaged with a 10× objective and quantified using ImageJ. **(A–E)**, Fluorescence images of worms at 3 days of adult age, expressing SOD-3::GFP, after exposures as indicated. **(F–J)**, Fluorescence images of worms at 3 days of adult age, expressing PEPCK::GFP, after exposures as indicated. Bars in images indicate 200 μm. Note that the SOD-3::GFP images are shown at slightly greater magnification than the PEPCK::GFP images. **(K)**, Histogram of mean ± SEM fluorescence for 10–20 worms per group, expressing SOD-3::GFP. **(L)**, Histogram of mean ± SEM fluorescence for 10–20 worms per group, expressing PEPCK::GFP. Statistical significances indicated for specific inter-group comparisons were calculated by two-tailed *t*-tests without adjustment for multiple comparisons.

DAF-16 target genes such as those encoding SOD-3 and PEPCK are strikingly upregulated in *age-1*(*mg44*) worms, and somewhat less so in *age-1*(*hx546*) mutants – increases that are blocked when *daf-16* is deleted (Shmookler Reis et al., 2009; Tazearslan et al., 2009). The DAF-16 transcription factor was shown to mediate strong modulation of expression (by factors of 2 to >1000) by the *age-1* allele for a total of 64 genes (Ayyadevara et al., 2009; Tazearslan et al., 2009). SOD-3 is an Fe^++^/Mn^++^ superoxide dismutase, believed to be mitochondrial and to protect against superoxide produced *via* the electron-transport chain; its mRNA is upregulated >9-fold in *age-1*(*mg44*)-F2 worms (Tazearslan et al., 2009). If PIA treatment, like *age-1* mutations, disrupts insulinlike signaling through DAF-16 activation, it should enhance transcription of *sod-3*. We treated young adult worms expressing a SOD-3::GFP fusion protein with several PIAs, each at 1 μM concentration. PIA24 increased SOD-3::GFP levels by 26% after 48 h (*P* < 0.0002), chiefly affecting diffuse global expression, whereas PIA6 and PIA12 produced increases of only 9% (*P* < 0.05) and 6% (not significant) respectively, while LY294002 slightly reduced fluorescence (Figure 7, panels A–E,K). We tested putative PI3K inhibitors for effects on *pck-2*, the *daf-16* target gene encoding phosphoenolpyruvate carboxykinase (PEPCK), which is upregulated >8.5-fold in *age-1*(*mg44*)-F2 worms (Tazearslan et al., 2009). PEPCK is a key enzyme of metabolic regulation which extends lifespan and increases physical activity levels as well as metabolic rate when overexpressed in mice (Hakimi et al., 2007). Adult worms expressing a PEPCK::GFP fusion protein were treated with 1 μM LY294002 or PIAs for 48 h. LY294002 increased PEPCK::GFP fluorescence by 25% (*P* < 0.01 relative to PIA7 controls), while PIAs 6, 12, and 24 elicited increases of 39–41% (each *P* < 0.002; Figure 7, panels F–J,L).

## Discussion

Since *age-1*(*mg44*) F2-homozygous worms show no full-length PI3K catalytic subunit, it does not appear to be synthesized by either alternative-splicing or read-through routes that avoid the mutationally introduced stop codon. We note that the expression of other components of insulin/IGF-1 signaling are also suppressed at the transcript level in this strain, as are catalytic subunits for all three classes of PI3K (Tazearslan et al., 2009). This underscores the point that the unique survival phenotypes associated with this mutant may not derive entirely from direct effects of the *age-1* nonsense mutation, but may instead be indirect consequences.

These worms evidence no measurable PI3K activity, even when strongly induced by peroxide stress in a transiently starved state. Although measures of PI3K activity averaged zero, such negative findings (no detectable class-I PI3K enzyme or activity) are constrained by the limited sensitivity of the assays, to the rather weak conclusion that F2 *mg44*^−/−^ worms have significantly less than half or one-fifth as much activity as wild-type (Figure 1C, left and right panels, respectively). In the PIP_3_ immunoassay, however, the F2 mutant level had such a small variance that it was significantly below 1.4% of the wild-type value. In contrast, their “F1” homozygous parents had 40% of wild-type PIP_3_ levels, which was far more than F2 progeny possessed (*P* < 10^−15^). These results are consistent with the hypothesis of maternal protection, wherein F1-homozygous worms acquire significant amounts of *age-1* mRNA, AGE-1 protein, and/or PIP_3_, from the oocytes formed in their heterozygous mothers. The *hx546* worms show higher levels of PIP_3_ than *age-1* F1 or F2 worms, suggesting that they retain substantial kinase activity despite our failure to detect any by PI3K activity assay (Figure 3). Given that strong *age-1* mutation indirectly suppresses transcription of *pten*, encoding the PTEN phosphatase that opposes AGE-1/PI3K (Tazearslan et al., 2009), it is possible for steady-state PIP_3_ levels to be reduced far less, in either *age-1* mutant, than PI3K itself.

We next determined the downstream consequences of chemical treatments thought to disrupt insulin/IGF-1 signaling, by monitoring global expression of SOD-3::GFP and PEPCK::GFP transgenic fusion proteins. The three active PIAs were of similar efficacy in stimulating PEPCK::GFP expression (Figure 7), and each was more effective than LY294002. When SOD-3::GFP fluorescence was assessed, however, LY294002 was actually inhibitory, and PIA24 was substantially more effective than either of the other PIAs. Since both *sod-3* and *pepck* are targets of the same FOXO transcription factor, DAF-16, it appears incongruous that the spectrum of drug activities should differ for these two endpoints. However, the tissue distribution differs between SOD-3 (globally expressed, with highest expression at the anterior tip, followed by the nerve ring just posterior to that, very similar to WormBase Expr8145 and Expr3925) and PEPCK (PCK-2, Expr6502 in WormBase: seen in adult intestine, reproductive organs, and vulval muscle, with lower expression in body-wall muscle); see Figure 7. Perhaps of even greater importance is the likelihood of multiple drug targets which differ among the PIAs (Gills et al., 2007). Differences between PIAs and more conventional PI3K inhibitors such as LY294002 are also expected, since PIAs were designed to target the PIP_3_-binding site of AKT [and may also target other PIP_3_-binding sites (Gills et al., 2007) including that of PI3K], whereas LY294002, wortmannin, and many other PI3K inhibitors, instead target the ATP-binding pocket of the PI3K catalytic subunit (Walker et al., 2000). It is thus quite possible that “collateral targets” of these drugs differ among tissues, and some of these targets may interact differentially with SOD-3 vs. PEPCK reporters. We note that such unintended drug targets might include effects on translation and protein turnover components, which could then influence expression of reporters.

These same drugs also varied with respect to protection of N2 adults from peroxide stress (Figure 6). For a given synthesis, they followed the same rank order (PIA24 > PIA6 > PIA12) as lifespan and the induction of SOD-3, which contributes to oxidant protection. The *age-1*(*hx546*) mutation only blunted the protection by PIA24 to approximately the same level as PIA12 (Figure 6B), which may be explicable if PIA24 confers part of its oxidative stress resistance through interaction with AGE-1, whereas the other PIAs act entirely *via* AGE-1-independent mechanisms (e.g., interaction with AKTs). LY294002 provided little or no benefit to either lifespan or peroxide survival; indeed, of all the drugs (other than PIAs) previously reported to inhibit PI3K, only GDC-0941 produced a nominally significant increase in *C. elegans* longevity, i.e., *P* < 0.03 without adjustment for multiple endpoints (Table 1). The absence of any short-term toxicity (or increased susceptibility to peroxide stress), for PIAs across a 1000-fold dose range, argues against a mechanism involving hormesis *via* deleterious drug effects. It remains to be seen whether drugs can be developed with greater specificity for class-I PI3K catalytic subunit, and whether those drugs will better recapitulate the extreme longevity and oxidative stress resistance of strong *age-1*-null mutations.

## Materials and Methods

### Strains

Nematode strains were supplied by the *Caenorhabditis* Genetics Center (CGC, Minneapolis) and were maintained at 20°C on 0.6% peptone NGM-agar plates seeded with *E. coli* strain OP50, as described (Ebert et al., 1993; Ayyadevara et al., 2001; Shmookler Reis et al., 2007). Cohorts were synchronized by alkaline hypochlorite lysis of parents (sparing eggs/larvae), and propagated on fresh nutrient-agar plates (Sulston and Hodgkin, 1988).

### Determination of life span

Nematodes, grown on NGM-agar plates containing 0.6% peptone, were harvested by rinsing off each plate with S buffer (0.1 M NaCl, 0.05 M potassium phosphate, pH 6.0) (Sulston and Hodgkin, 1988). Adults were allowed to settle, and then resuspended in alkaline hypochlorite (0.5 N NaOH, 1.05% hypochlorite; 5 min at 20°C). The recovered eggs (containing unenclosed larvae) were rinsed in S buffer and placed on fresh agar plates seeded with *E. coli* strain OP50. Survival cultures were established on 60-mm NGM-agar plates (Sulston and Hodgkin, 1988; Ebert et al., 1993; Ayyadevara et al., 2003) seeded with OP50. PI3K inhibitors, including LY294002, wortmannin, A66, ZSTK474, and GDC-0941 (Selleckchem, Houston, TX, USA) or phosphatidylinositol analogs (PIAs, from A. Kozikowski, University of Illinois, Chicago) dissolved in DMSO, were overlaid on plates to achieve final concentrations of 1, 2, or 10 μM. Worms were added 6 h later, 1 day after the L4/adult molt; 30–50 adults were transferred to each 60-mm dish. Worms were maintained at 20°C, and scored as alive, dead, or lost during daily transfer to fresh dishes. Worms were considered dead if they did not move either spontaneously or in response to touch; those lost (stranded on dish walls or beneath the agar) or killed by internal hatching of progeny (“bagging”) were censored at the midpoint of the time interval in which this occurred; worms inadvertently killed were censored at the time of the event.

### Hydrogen peroxide stress tolerance after PIA treatment

Adult worms [N2DRM or *age-1*(*hx546*), as indicated] were synchronized by alkaline hypochlorite lysis of hermaphrodites; surviving embryos were transferred to fresh NGM-agar plates and allowed to mature. On reaching the L4 stage, worms were placed on fresh NGM-agar plates seeded with OP50 and overlaid with PIAs to achieve 1 μM. After 48 h, they were transferred to 24-well plates (20–25 worms per well) containing S medium (S buffer plus 0.5% cholesterol) and 5- or 7-mM hydrogen peroxide (Sigma) at 20°C, as previously described (Ayyadevara et al., 2005, 2008). Survival was scored as above, initially at 1-h intervals, until no worms remained alive. Assays, each comprising 20 or 40 worms, were performed two to six times per strain.

### PIP_3_ staining and immunofluorescence

N2DRM, *age-1*(*hx546*), and *age-1*(*mg44*) worms were fixed at indicated ages by a modified Finney–Ruvkun protocol (Finney and Ruvkun, 1990). Briefly, worms were rinsed from plates in S buffer, rinsed again, and fixed at room temperature in S buffer containing 1% formaldehyde. The solution was frozen on dry ice and thawed overnight at 4°C with slow agitation. After centrifugation, 30 s at 2000 rpm, supernatant was aspirated and worms were washed twice with Tris-Triton buffer (“TTB,” comprising 0.1 M Tris-Cl, pH 7.4; 10 mM EDTA; and 1% v/v Triton X-100), then incubated 2.5 h at 37°C in TTB plus 1% w/v β-mercaptoethanol to reduce disulfide bonds. Worms were washed twice in 25-mM borate buffer, pH 9.2, incubated 15 min at 37°C with borate plus 10- mM DTT, and exposed 15 min at 20°C to 0.3% H_2_O_2_ in borate buffer. After rinsing in antibody B buffer [0.14 M NaCl; 50-mM sodium phosphate buffer, pH 7.9; 0.1% w/v bovine serum albumin (BSA); 0.1% v/v Triton X-100; 10-mM EDTA; and 0.05% w/v NaN_3_], they were incubated at 4°C overnight with mouse IgM antibody to PI_(3,4,5)_P_3_ (Echelon Biosciences Inc.) diluted 1:50 in antibody A buffer (identical to B buffer but with 1% w/v BSA). Worms were washed in antibody B buffer (3 min × 30 min), then incubated 1.5 h at room temperature with ALEXA594-labeled donkey anti-mouse IgG (Molecular Probes) diluted 1:200 in antibody A buffer. After three 30-min washes in antibody B buffer, worms were counterstained with DAPI (Invitrogen) and mounted on slides with Prolong Gold Antifade Reagent (Invitrogen); images were captured with an Olympus BX51 fluorescence microscope.

### Expression of SOD-3::GFP and PEPCK::GFP followed by PIA treatment

PIAs were dissolved in DMSO, creating 20× stocks that produce final PIA concentrations of 1 or 10 μM in the agar plates. These solutions were overlaid on 60-mm dishes previously seeded with *E. coli* strain OP50. Adult worms were of strain TJ374 (zEx374) expressing SOD-3::GFP fusion protein, or strain BC10543 (sEx10543) expressing a PCK-2(R11A5.4)::GFP fusion protein. They were synchronized by alkaline hypochlorite lysis of hermaphrodites, and surviving embryos were transferred to fresh NGM-agar plates. Young adults (post-L4 molt) were transferred to NGM-agar plates with PIAs. After 48 h, adult worms were collected, washed three times in S buffer, and fixed in 1% formaldehyde. Worms were mounted on slides under a coverslip, and fluorescence images were taken using an Olympus BX51 microscope.

### *In vivo* PIP_3_ assay

Wild-type and *age-1* mutant worms were collected and washed in phosphate-free RPMI-1640 medium, then labeled at 20°C with 1 mCi/ml [^32^P]-orthophosphate for 16 h in phosphate-free RPMI-1640 medium. ^32^P-labeled worms were then washed once in phosphate-free RPMI-1640 medium, and lipids were extracted and separated by thin-layer chromatography according to a published procedure (Weinkove et al., 2006). After autoradiography, spots were scraped from the chromatograph and β emissions quantified in a scintillation counter (Beckman).

## Conflict of Interest Statement

The authors declare that the research was conducted in the absence of any commercial or financial relationships that could be construed as a potential conflict of interest.
